# Polymorphisms of *VDR* gene and risk of gastric cardiac adenocarcinoma in Chinese population

**DOI:** 10.18632/oncotarget.17270

**Published:** 2017-04-20

**Authors:** Jun Yin, Huiwen Pan, Tao Long, Lu Lv, Peng Zhai, Chao Liu, Aizhong Shao, Yijun Shi, Yangyong Sun, Jingfeng Zhu, Liming Wang, Guowen Ding, Suocheng Chen, Weifeng Tang, Cheng Qian, Lijie Tan, Haiyong Gu

**Affiliations:** ^1^ Department of Cardiothoracic Surgery, Affiliated People's Hospital of Jiangsu University, Zhenjiang, China; ^2^ Department of Chemotherapy, Cancer Institute, Affiliated People's Hospital of Jiangsu University, Zhenjiang, China; ^3^ Department of Thoracic Surgery, Zhongshan Hospital of Fudan University, Shanghai, China

**Keywords:** VDR polymorphisms, gastric cardiac adenocarcinoma, association

## Abstract

*Vitamin D receptor* (*VDR*) gene polymorphisms have been reported to increase susceptibility to some malignant tumors, yet the effect on gastric cardiac adenocarcinoma susceptibility remains unknown. Here, we conducted a hospital-based case-control study to examine the correlation of single nucleotide polymorphisms of *VDR* rs2107301T>C, rs2228570C>T, rs1989969C>T and rs11568820 G>A and gastric cardiac adenocarcinoma susceptibility. A total 330 cases and 608 controls were enrolled in the study. Using ligation detection reaction, we found that the variant alleles of the four polymorphisms were not associated with risk of gastric cardiac adenocarcinoma. Further stratified analyses showed that there was an increased risk associated with *VDR* rs1989969 polymorphism among patients who were drinking or aged <60. The haplotypes VDR T_rs2107301_T_rs2228570_C_rs1989969_G_rs11568820_ reduced the susceptibility. This study demonstrated that *VDR* rs1989969 polymorphism was involved in the carcinogenesis of gastric cardiac adenocarcinoma, especially increased the risk in the younger and alcohol drinking Chinese population.

## INTRODUCTION

Gastric cardia adenocarcinoma (GCA) remains one of the most common malignant tumor worldwide [1]. Although the incidence of noncardia gastric cancer has declined steadily, the incidence and mortality of GCA are continuously increasing [2]. The etiology of GCA comprises interactions of multiple environmental and genetic factors. Environmental factors including cigarette smoking and alcohol consumption [3, 4], as well as genetic factors such as gene polymorphisms, have been implicated with GCA. Yet, the underlying etiological mechanisms of GCA are not fully understood.

The 1,25-dihydroxyvitamin D_3_ [1,25(OH)_2_D_3_] is the hormonally active form of vitamin D, which has been shown to inhibit prostate, breast and colon cancer cell progression [5]. Consistently, amounting evidence has indicated the correlation of the Vitamin D Receptor (VDR) and cancer. The antineoplastic effect of 1,25(OH)_2_D_3_ requires the expression of VDR in tumor cells [6]. Increased tumor VDR expression is associated with a better prognosis in various types of cancer [7, 8]. The association of single nucleotide polymorphisms (SNPs) in *VDR* (ApaI [rs7975232], BsmI [rs1544410], FokI [rs10735810], TaqI [rs731236] and cancer risk has been reported, yet the results were inconclusive [6, 9–12].

In our previous study, we have investigated the correlation of four SNPs *VDR* rs11568820 G>A, *VDR* rs1989969 C>T, *VDR* rs2107301 T>C and *VDR* rs2228570 C>T with esophageal squamous cell carcinoma development, and found that *VDR* rs2107301 T>C polymorphism with alcohol drinking enhanced the risk of esophageal squamous cell carcinoma [13]. Considering GCA occurs in the immediate vicinity of esophagus, we hypothesized that these four SNPs are also related to GCA. Here, we performed a hospital-based case-control study to examine the genetic effects of these four SNPs on the development of GCA.

## RESULTS

### Characteristics of the study subjects

The characteristics of the study subjects, including demographics and environmental risk factors, are presented in Table 1. The cases and controls were well matched in gender and age (χ^2^ test, *p*=0.746 and 0.965, respectively). However, tobacco smoking rate was much higher in GCA patients as compared with the control subjects (36.67% vs. 27.96%, *p*=0.006). Alcohol drinking rate was higher, yet not significantly, in GCA patients than in control subjects (29.39% vs. 23.01%, *p*=0.072).

**Table 1:** Distribution of selected demographic variables and risk factors in GCA cases and controls

Variable	Cases (n = 330)		Controls (n = 608)		*p*^a^
	n	%	n	%	
Age (years) mean ± SD	65.06 (±8.37)		64.19 (±6.66)		0.103
Age (years)					0.746
< 60	89	26.97	170	27.96	
≥ 60	241	73.03	438	72.04	
Sex					0.965
Male	223	67.58	410	67.43	
Female	107	32.42	198	32.57	
Tobacco use					**0.006**
Never	209	63.33	438	72.04	
Ever	121	36.67	170	27.96	
Alcohol use					0.072
Never	233	70.61	462	75.99	
Ever	97	29.39	146	24.01	

^a^ Two-sided χ^2^ test and student t test; Bold values are statistically significant (*p* <0.05).

As shown in Table 2, the genotyping successful rates were ranging from 95.76% to 100.0% in GCA cases and from 95.39 to 99.18% in controls. Compared with the minor allele frequency (MAF) for Chinese in database for all four SNPs loci, the MAF in our controls was similar (Table 2). In the control subjects, the genotype frequencies for these four polymorphisms reached Hardy-Weinberg equilibrium (*p*-value for HWE, all *p*>0.05, Table 2).

**Table 2:** Primary information for *VDR* rs2107301 T>C, rs2228570 C>T, rs1989969 C>T and rs11568820 G>A polymorphisms

Genotyped SNPs	*VDR* rs2107301 T>C	*VDR* rs2228570 C>T	*VDR* rs1989969 C>T	*VDR* rs11568820 G>A
Chromosome	12	12	12	12
Gene (ID)	VDR (7421)	VDR (7421)	VDR (7421)	VDR (7421)
Function	Intron region	Missense	Intron region	Intergene region
Chr Pos (Genome Build 36.3)	46541837	46559162	46564277	46588812
Regulome DB Score^a^	5	5	No data	No data
TFBS^b^	—	—	—	Y
Splicing (ESE or ESS)	—	Y	—	—
nsSNP	—	Y	—	—
MAF^c^ for Chinese in database	0.291	0.482	0.330	0.453
MAF in our controls (n = 608)	0.297	0.456	0.323	0.433
*p* value for HWE^d^ test in our controls	0.690	0.347	0.718	0.574
Genotyping method^e^	LDR	LDR	LDR	LDR
% Genotyping value	95.52%	95.52%	98.19%	98.08%

^a^ http://www.regulomedb.org/;

^b^ TFBS: Transcription Factor Binding Site (http://snpinfo.niehs.nih.gov/snpinfo/snpfunc.htm);

^c^ MAF: minor allele frequency;

^d^ HWE: Hardy–Weinberg equilibrium;

^e^ LDR: ligation detection reaction.

### Associations between risk of GCA and four polymorphisms

As demonstrated in Table 3, the single locus analyses showed no statistically significant difference in genotype frequencies of four SNPs between the cases and the controls (*p*>0.05). There are no correlation between these four polymorphic sites with the risk of GCA as evaluated by the logistic regression analyses (Table 3).

**Table 3:** Logistic regression analyses of associations between *VDR* rs2107301 T>C, rs2228570 C>T, rs1989969 C>T and rs11568820 G>A polymorphisms and risk of GCA

Genotype	Cases (n = 330)		Controls (n = 608)		Crude OR (95%CI)	*p*	Adjusted OR ^a^ (95%CI)	*p*
	n	%	n	%				
*VDR* rs2107301 T>C								
TT	155	49.05	285	49.14	1.00		1.00	
TC	129	40.82	246	42.41	0.96 (0.72-1.29)	0.805	0.97 (0.72-1.29)	0.819
CC	32	10.13	49	8.45	1.20 (0.74-1.95)	0.461	1.19 (0.73-1.95)	0.492
CC vs. TC vs. TT								0.683
TC+CC	161	50.95	295	50.86	1.00 (0.76-1.32)	0.980	1.00 (0.76-1.32)	0.982
TT+TC	284	89.87	531	91.55	1.00		1.00	
CC	32	10.13	49	8.45	1.22 (0.77-1.95)	0.403	1.21 (0.75-1.94)	0.436
C allele	193	30.54	344	29.66				
*VDR* rs2228570 C>T								
CC	97	30.70	166	28.62	1.00		1.00	
CT	153	48.42	299	51.55	0.88 (0.64-1.20)	0.412	0.89 (0.65-1.23)	0.488
TT	66	20.89	115	19.83	0.98 (0.66-1.46)	0.928	0.97 (0.65-1.45)	0.895
TT vs. CT vs. CC								0.666
CT+TT	219	69.30	414	71.38	0.91 (0.67-1.22)	0.515	0.92 (0.68-1.24)	0.567
CC+CT	250	79.11	465	80.17	1.00		1.00	
TT	66	20.89	115	19.83	1.07 (0.76-1.50)	0.706	1.05 (0.74-1.47)	0.799
T allele	285	45.09	529	45.60				
*VDR* rs1989969 C>T								
CC	135	42.45	278	46.10	1.00		1.00	
CT	140	44.03	260	43.12	1.11 (0.83-1.48)	0.486	1.10 (0.82-1.48)	0.521
TT	43	13.52	65	10.78	1.36 (0.88-2.11)	0.165	1.36 (0.88-2.11)	0.172
TT vs. CT vs. CC								0.369
CT+TT	183	57.55	325	53.90	1.16 (0.88-1.53)	0.290	1.15 (0.87-1.52)	0.314
CC+CT	275	86.48	538	89.22	1.00		1.00	
TT	43	13.52	65	10.78	1.29 (0.86-1.95)	0.220	1.30 (0.86-1.96)	0.221
T allele	226	35.53	390	32.34				
*VDR* rs11568820 G>A								
GG	99	30.00	193	32.71	1.00		1.00	
GA	162	49.09	283	47.97	1.12 (0.82-1.52)	0.488	1.11 (0.81-1.53)	0.500
AA	69	20.91	114	19.32	1.18 (0.80-1.73)	0.399	1.20 (0.81-1.77)	0.361
AA vs. GA vs. GG								0.666
GA+AA	231	70.00	397	67.29	1.13 (0.85-1.52)	0.397	1.14 (0.85-1.53)	0.390
GG+GA	261	79.09	476	80.68	1.00		1.00	
AA	69	20.91	114	19.32	1.10 (0.79-1.54)	0.563	1.12 (0.80-1.58)	0.503
A allele	300	45.45	511	43.31				

^a^ Adjusted for age, sex, smoking status and alcohol consumption.

### Stratification analyses of four polymorphisms and risk of GCA

To further assess the effects of *VDR* rs11568820 G>A, rs1989969 C>T, rs2107301 T>C and rs2228570 C>T on GCA risk according to different sex, smoking, age and alcohol drinking status, stratification analyses were conducted as shown in Tables 4, 5, 6, 7, respectively. In association with the *VDR* rs1989969 C>T polymorphism, we further identified two significantly increased risk factors of GCA, which are age<60 or alcohol drinking.

**Table 4:** Stratified analyses between *VDR* rs1989969 C>T polymorphism and GCA risk by sex, age, smoking status and alcohol consumption

Variable	*VDR* rs1989969 C > T (case/control)^a^				Adjusted OR^b^ (95% CI); *p*; *p*_h_^c^				
	CC	CT	TT	CT+TT	CC	CT	TT	CT+TT	TT vs. (CT+CC)
Sex									
Male	92/189	98/170	28/46	126/216	1.00	1.19 (0.83-1.69); *p*: 0.345; *p*_h_:0.523	1.26 (0.74-2.14); *p*: 0.405; *p*_h_:0.575	1.20 (0.86-1.68); *p*: 0.283; *p*_h_:0.734	1.15 (0.70-1.91); *p*: 0.579; *p*_h_:0.412
Female	43/89	42/90	15/19	57/109	1.00	1.00 (0.59-1.70); *p*: 0.999; *p*_h_:0.523	1.67 (0.76-3.66); *p*: 0.199; *p*_h_:0.575	1.12 (0.68-1.84); *p*: 0.660; *p*_h_:0.734	1.67 (0.80-3.50); *p*: 0.172; *p*_h_:0.412
Age									
<60	31/90	42/65	15/14	57/79	1.00	1.85 (1.03-3.33); ***p*: 0.041; *p*_h_:0.031**	2.97 (1.24-7.13); ***p*: 0.015; *p*_h_:0.023**	2.05 (1.18-3.57); ***p*: 0.011; *p*_h_:0.010**	2.19 (0.97-4.97); *p*: 0.060; *p*_h_:0.096
≥60	104/188	98/195	28/51	126/246	1.00	0.90 (0.64-1.27); *p*: 0.560; *p*_h_:0.031	0.99 (0.59-1.67); *p*: 0.977; *p*_h_:0.023	0.92 (0.67-1.27); *p*: 0.621; *p*_h_:0.010	1.04 (0.64-1.71); *p*: 0.865; *p*_h_:0.096
Smoking status									
Never	88/197	87/189	25/48	112/237	1.00	1.01 (0.70-1.46); *p*: 0.962; *p*_h_:0.482	1.12 (0.64-1.97); *p*: 0.696; *p*_h_:0.345	1.03 (0.73-1.46); *p*: 0.863; *p*_h_:0.359	1.11 (0.65-1.90); *p*: 0.692; *p*_h_:0.451
Ever	47/81	53/71	18/17	71/88	1.00	1.24 (0.74-2.10); *p*: 0.415; *p*_h_:0.482	1.90 (0.87-4.15); *p*: 0.107; *p*_h_:0.345	1.37 (0.84-2.24); *p*: 0.213; *p*_h_:0.359	1.71 (0.82-3.56); *p*: 0.154; *p*_h_:0.451
Alcohol consumption									
Never	99/205	93/206	30/47	123/253	1.00	0.90 (0.63-1.27); *p*: 0.538; *p*_h_:0.057	1.31 (0.77-2.23); *p*: 0.315; *p*_h_:0.835	0.97 (0.70-1.35); *p*: 0.865; *p*_h_:0.100	1.39 (0.84-2.28); *p*: 0.202; *p*_h_:0.647
Ever	36/73	47/54	13/18	60/72	1.00	1.90 (1.04-3.46); ***p*: 0.036; *p*_h_:0.057**	1.45 (0.60-3.50); *p*: 0.409; *p*_h_:0.835	1.78 (1.01-3.14); ***p*: 0.045; *p*_h_:0.100**	1.05 (0.46-2.40); *p*: 0.903; *p*_h_:0.647

^a^ The genotyping was successful in 318 (96.36%) GCA cases and 603 (99.18%) controls for *VDR* rs1989969 C>T;

^b^ Adjusted for age, sex, smoking status and alcohol consumption (besides stratified factors accordingly) in a logistic regression model;

^c^ *p*_h_ for heterogeneity; Bold values are statistically significant (*p* <0.05).

**Table 5:** Stratified analyses between *VDR* rs2228570 polymorphism and GCA risk by sex, age, smoking status and alcohol consumption

Variable	*VDR* rs2228570 (case/control)^a^				Adjusted OR^b^ (95% CI); *p*; *p*_h_^c^				
	CT	CC	TT	CT+TT	TT	CT	CC	CT+CC	CC vs. (CT+TT)
Sex									
Male	101/205	71/113	40/73	141/278	1.00	1.112 (0.71-1.71); *p*: 0.646; *p*_h_:0.572	0.872 (0.54-1.42); *p*:0.581; *p*_h_:0.559	1.013 (0.66-1.55); *p*:0.953; *p*_h_:0.965	0.807 (0.56-1.16); *p*: 0.242; *p*_h_:0.984
Female	52/94	26/53	26/42	78/136	1.00	0.894 (0.49-1.62); *p*:0.711; *p*_h_: 0.572	0.792 (0.40-1.56); *p*:0.501; *p*_h_:0.559	0.857 (0.49-1.50); *p*:0.590; *p*_h_:0.965	0.855 (0.49-1.48); *p*: 0.574; *p*_h_:0.984
Age									
<60	45/78	25/48	15/33	60/111	1.00	1.269 (0.62-2.58); *p*:0.511; *p*_h_:0.945	1.146 (0.53-2.49); *p*: 0.732; *p*_h_:0.245	1.222 (0.62-2.40); *p*:0.561; *p*_h_:0.746	1.038 (0.58-1.85); *p*:0.900; *p*_h_:0.741
≥60	108/221	72/118	51/82	159/303	1.00	0.786 (0.52-1.19); *p*:0.258; *p*_h_:0.945	0.981 (0.62-1.55); *p*: 0.935; *p*_h_:0.245	0.854 (0.58-1.27); *p*: 0.431; *p*_h_:0746	1.163 (0.82-1.65); *p*: 0.399; *p*_h_:0.741
Smoking status									
Never	108/221	69/117	45/78	153/299	1.00	0.847 (0.55-1.31); *p*:0.452; *p*_h_:0.062	1.022 (0.64-1.64); *p*:0.927; *p*_h_:0.722	0.908 (0.60-1.37); *p*:0.643; *p*_h_:0.788	0.868 (0.61-1.24); *p*:0.43; *p*_h_:0.200
Ever	45/78	28/49	21/37	66/115	1.00	1.016 (0.53-1.95); *p*:0.961; *p*_h_:0.062	1.007 (0.49-2.04); *p*:0.985; *p*_h_:0.722	1.013 (0.55-1.86); *p*:0.967; *p*_h_:0.788	1.004 (0.58-1.75); *p*:0.988; *p*_h_:0.200
Alcohol consumption									
Never	122/228	72/125	50/86	172/314	1.00	0.920 (0.61-1.39); *p*:0.693; *p*_h_:0.051	0.991(0.63-1.56); *p*:0.968; *p*_h_:0.893	0.945(0.64-1.39); *p*:0.777; *p*_h_:0.513	0.951 (0.67-1.34); *p*:0.775; *p*_h_:0.143
Ever	31/71	25/41	16/29	47/100	1.00	0.791 ( 0.37-1.66); *p*:0.536;*p*_h_:0.051	1.105 (0.50-2.43); *p*:0.803; *p*_h_:0.893	0.906 (0.45-1.80); *p*:0.78; *p*_h_:0.513	0.771 (0. 42-1.41); *p*:0.399; *p*_h_:0.143

^a^ The genotyping was successful in 316 (95.75%) GCA cases and 603 (95.70%) controls *VDR* rs2228570 C>T;

^b^ Adjusted for age, sex, smoking status and alcohol consumption (besides stratified factors accordingly) in a logistic regression model;

^c^ *p*_h_ for heterogeneity; bold values are statistically significant (*p* <0.05).

**Table 6:** Stratified analyses between *VDR* rs2107301 polymorphism and GCA risk by sex, age, smoking status and alcohol consumption

Variable	*VDR* rs2107301 (case/control)^a^				Adjusted OR^b^ (95% CI); *p*; *p*_h_^c^				
	CC	CT	TT	CT+TT	CC	CT	TT	CT+TT	TT vs. (CT+CC)
Sex									
Male	21/30	86/174	105/187	191/361	1.00	0.706 (0.38-1.31); *p*:0.266; *p*_h_:0.947	0.802 (0.437-1.47); *p*:0.476; *p*_h_:0.510	0.756 (0.421-1.36); *p*:0.756; *p*_h_:0.983	0.934 (0.669-1.31); *p*:0.669; *p*_h_:0.995
Female	11/19	43/72	50/98	93/170	1.00	1.032 (0.45-2.37); *p*:0.942; *p*_h_: 0.947	0.881 (0.39-1.99); *p*:0.762; *p*_h_: 0.510	0.945 (0.43-2.07); *p*:0.887; *p*_h_: 0.983	1.163 (0.72-1.88); *p*:0.536; *p*_h_:0.995
Age									
<60	9/15	36/71	40/73	76/144	1.00	0.845 (0.34-2.12); *P*:0.719; *p*_h_: 0.607	0.913 (0.37-2.27); *p*:0.845; *p*_h_:0.784	0.880 (0.37-2.1); *p*:0.773; *p*_h_:0.735	0.955 (0.56-1.62); *p*:0.864; *p*_h_:0.746
≥60	23/34	93/175	115/212	208/387	1.00	0.786 (0.44-1.41); *p*:0.419; *p*_h_0.607	0.802 (0.45-1.43); *p*:0.452; *p*_h_: 0.784	0.795 (0.456-1.39); *p*:0.416; *p*_h_: 0.735	1.023 (0.74-1.41); *p*:0.889; *p*_h_:0.746
Smoking status									
Never	24/36	94/176	104/204	198/380	1.00	0.801 (0.45-1.42); *p*:0.448; *p*_h_:0.753	0.765 (0.433-1.35); *p*:0.354; *p*_h_:0.248	1.279 (0.74-2.21); *p*:0.374; *p*_h_:0.216	1.092 (0.78-1.51); *p*:0.598; *p*_h_:0.205
Ever	8/13	35/70	51/81	86/151	1.00	0.813 (0.31-2.14); *p*:0.674; *p*_h_: 0.753	1.023 (0.397-2.64); *p*:0.962; *p*_h_: 0.248	0.925 (0.37-2.32); *p*:0.869; *p*_h_:0.216	0.823 (0.49-1.37); *p*:0.452; *p*_h_:0.205
Alcohol consumption									
Never	25/42	103/189	116/208	219/397	1.00	0.916 (0.53-1.58); *p*:0.753; *p*_h_:0.959	0.937 (0.54-1.62); *p*:0.815; *p*_h_:0.204	0.927 (0.55-1.56); *p*:0.775; *p*_h_:0.159	0.994 (0.73-1.36); *p*:0.968; *p*_h_:0.147
Ever	7/7	26/57	39/77	65/134	1.00	0.456 (0.15-1.43); *p*:0.172; *p*_h_: 0.959	0.506 (0.166-1.55); *p*:0.226; *p*_h_: 0.204	0.485 (0.16-1.44); *p*:0.18; *p*_h_:0.159	1.018 (0.57-1.80); *p*:0.951; *p*_h_:0.147

^a^ The genotyping was successful in 316 (95.75%) GCA cases and 603 (95.39%) controls for *VDR* rs1989969 C>T;

^b^ Adjusted for age, sex, smoking status and alcohol consumption (besides stratified factors accordingly) in a logistic regression model;

^c^ *p*_h_ for heterogeneity; bold values are statistically significant (*p* <0.05).

**Table 7:** Stratified analyses between *VDR* rs11568820 polymorphism and GCA risk by sex, age, smoking status and alcohol consumption

Variable	*VDR* rs11568820 (case/control)^a^				Adjusted OR^b^ (95% CI); *p*; *p*_h_^c^				
	AA	AG	GG	AG+GG	AA	AG	GG	AG+GG	GG vs. (AG+AA)
Sex									
Male	49/85	100/184	74/126	174/310	1.00	0.943 (0.65-1.45); *p*: 0.787; *p*_h_:0.399	1.019 (0.65-1.60); *p*:0.936; *p*_h_:0.352	0.974(0.65-1.45); *p*:0.895; *p*_h_:0.927	0.943 (0.66-1.34); *p*:0.743; *p*_h_:0.895
Female	20/29	62/99	25/67	87/166	1.00	0.908 (0.47-1.74); *p*:0.772; *p*_h_: 0.399	0.541 (0.26-1.13); *p*:0.098; *p*_h_:0.352	0.760(0.41-1.42); *p*:0.389; *p*_h_:0.927	1.72 (1.00-2.94); *p*:0.057^e^; *p*_h_:0.895
Age									
<60	14/34	48/69	27/60	75/129	1.00	1.689 (0.82-3.48); *p*:0.153; *p*_h_:0.799	1.093 (0.51-2.36); *p*:0.821; *p*_h_:0.151	1.412 (0.71-2.79); *p*:0.322; *p*_h_:0.824	1.338 (0.77-2.33); *p*:0.302; *p*_h_:0.844
≥60	55/80	114/214	72/133	186/347	1.00	0.775 (0.51-1.17); *p*:0.224; *p*_h_: 0.799	0.787 (0.50-1.23); *p*:0.295; *p*_h_:0.151	0.780 (0.53-1.15); *p*:0.207; *p*_h_:0.824	1.062 (0.75-1.49); *p*:0.732; *p*_h_:0.844
Smoking status									
Never	50/83	120/208	65/135	185/343	1.00	0.958 (0.63-1.45); *p*:0.839; *p*_h_:0.214	0.799 (0.51-1.26); *p*:0.338; *p*_h_:0.097	0.895 (0.60-1.33); *p*:0.582; *p*_h_:0.200	1.213 (0.85-1.72); *p*:0.280; *p*_h_:0.191
Ever	19/31	42/75	34/58	76/133	1.00	0.914 (0.46-1.81); *p*:0.796; *p*_h_:0.214	0.956 (0.47-1.95); *p*:0.902; *p*_h_:0.097	0.932 (0.49-1.76); *p*:0.829; *p*_h_:0.200	0.982 (0.58-1.66); *p*:0.945; *p*_h_:0.191
Alcohol consumption									
Never	58/84	126/220	73/145	199/365	1.00	0.829 (0.56-1.24); *p*:0.359; *p*_h_:0.176	0.729 (0.47-1.13); *p*:0.156; *p*_h_:0.060	0.790 (0.54-1.15); *p*:0.218; *p*_h_:0.150	1.202 (0.86-1.68); *p*:0.282; *p*_h_:0.145
Ever	11/30	36/63	26/48	62/111	1.00	1.558 (0.69-3.48); *p*:0.277; *p*_h_:0.176	1.477 (0.64-3.42); *p*:0.361; *p*_h_:0.060	1.523 (0.71-3.25); *p*:0.274; *p*_h_:0.150	0.933 (0.52-1.69); *p*:0.818; *p*_h_:0.145

^a^ The genotyping was successful in 330 (100%) GCA cases and 603 (97.04%) controls for *VDR* rs11568820 A>G;

^b^ Adjusted for age, sex, smoking status and alcohol consumption (besides stratified factors accordingly) in a logistic regression model;

^c^ *p*_h_ for heterogeneity; bold values are statistically significant (*p* <0.05).

### Linkage disequilibrium analyses

Linkage disequilibrium analyses in both controls and cases were conducted as shown in Table 8. *D*’ and *r*^2^ were analyzed and showed that there were weak correlations between the four loci.

**Table 8:** Linkage disequilibrium analyses of *VDR* rs2228570, rs1989969, rs11568820 and rs2107301 in control and case groups

	Control			Case		
	rs2228570	rs1989969	rs11568820	rs2228570	rs1989969	rs11568820
*D*’						
rs2107301	0.289	0.224	0.136	0.202	0.105	0.015
rs2228570	-	0.213	0.069	-	0.206	0.138
rs1989969	-	-	0.249	-	-	0.366
*r*^2^						
rs2107301	0.029	0.010	0.006	0.015	0.003	0.000
rs2228570	-	0.026	0.004	-	0.029	0.019
rs1989969	-	-	0.039	-	-	0.092

### Haplotype analysis of VDR polymorphisms and susceptibility of GCA

As shown in Table 9, haplotype analysis was also conducted and haplotypes were from the genotypes of *VDR* polymorphisms. The haplotype analysis indicated that the *VDR* T_rs2107301_C_rs2228570_C_rs1989969_G_rs11568820_ was the most common haplotype in both groups (17.27% in case group, 14.88% in control group). Compared with the haplotype T_rs2107301_C_rs2228570_C_rs1989969_G_rs11568820_, the haplotypes *VDR* T_rs2107301_T_rs2228570_C_rs1989969_G_rs11568820_ were more common in the controls (0.143) than in the case group (0.113) with significant difference (*p*=0.038). T_rs2107301_T_rs2228570_C_rs1989969_G_rs11568820_ haplotype was associated with a significantly reduced risk of GCA (OR=0.68, 95%CI=0.48-0.98, *p*=0.038). We also further conducted other haplotypes and risk of GCA, but no association was observed between patients and controls.

**Table 9:** *VDR* haplotype frequencies (%) in cases and controls and risk of GCA

Haplotypes	Cases (n = 660)		Controls (n = 1216)		Crude OR (95% CI)	*p*
	n	%	n	%		
*VDR* T_rs2107301_C_rs2228570_C_rs1989969_G_rs11568820_	114	17.27	181	14.88	1.00	
*VDR* T_rs2107301_T_rs2228570_C_rs1989969_G_rs11568820_	75	11.36	174	14.31	0.68 (0.48-0.98)	0.038
*VDR* T_rs2107301_C_rs2228570_C_rs1989969_A_rs11568820_	54	8.18	123	10.12	0.70 (0.47-1.04)	0.075
*VDR* T_rs2107301_T_rs2228570_T_rs1989969_A_rs11568820_	74	11.21	114	9.38	1.03 (0.71-1.50)	0.875
*VDR* C_rs2107301_C_rs2228570_C_rs1989969_G_rs11568820_	59	8.94	115	9.46	0.82 (0.55-1.21)	0.305
*VDR* T_rs2107301_T_rs2228570_C_rs1989969_A_rs11568820_	44	6.67	86	7.07	0.81 (0.53-1.25)	0.346
*VDR* C_rs2107301_C_rs2228570_C_rs1989969_A_rs11568820_	31	4.70	65	5.35	0.76 (0.47-1.23)	0.264
*VDR* T_rs2107301_C_rs2228570_T_rs1989969_G_rs11568820_	29	4.39	63	5.18	0.73 (0.44-1.20)	0.218
*VDR* T_rs2107301_C_rs2228570_T_rs1989969_A_rs11568820_	36	5.45	62	5.10	0.92 (0.58-1.48)	0.736
*VDR* T_rs2107301_T_rs2228570_T_rs1989969_G_rs11568820_	34	5.15	58	4.77	0.93 (0.57-1.51)	0.771
*VDR* C_rs2107301_T_rs2228570_C_rs1989969_G_rs11568820_	27	4.09	50	4.11	0.86 (0.51-1.45)	0.565
*VDR* C_rs2107301_C_rs2228570_T_rs1989969_G_rs11568820_	15	2.27	31	2.55	0.77 (0.40-1.49)	0.433
*VDR* C_rs2107301_T_rs2228570_C_rs1989969_A_rs11568820_	20	3.03	29	2.38	1.10 (0.59-2.03)	0.773
*VDR* C_rs2107301_T_rs2228570_T_rs1989969_A_rs11568820_	17	2.58	25	2.06	1.08 (0.56-2.09)	0.820
*VDR* C_rs2107301_C_rs2228570_T_rs1989969_A_rs11568820_	24	3.64	23	1.89	1.66 (0.89-3.07)	0.109
*VDR* C_rs2107301_T_rs2228570_T_rs1989969_G_rs11568820_	7	1.06	17	1.40	0.65 (0.26-1.63)	0.361

With the order of *VDR* rs2107301 T>C, rs2228570 C>T, rs1989969 C>T and rs11568820 G>A in gene position.

## DISCUSSION

In the current study, by multivariable logistic analysis, we demonstrated that there was no significant association between the polymorphisms of *VDR* rs11568820 G>A, rs1989969 C>T, rs2107301 T>C andrs2228570 C>T and the risk of GCA in Chinese population. Yet, notably, we detected an increased risk of GCA among alcohol drinking or younger patients (<60 of age) who carried *VDR* rs1989969 C>T genotype. Interestingly, the *VDR* T_rs2107301_T_rs2228570_C_rs1989969_G_rs11568820_ haplotype was associated with a significantly reduced risk of GCA.

Recently, accumulating evidence showed 1,25(OH)_2_D_3_, the hormonally active form of vitamin D, participates in apoptosis, cell proliferation and inflammation in cancer [5]. 1,25(OH)_2_D_3_ could restrain cancer cell growth by inducing their differentiation, by arresting cells in the G_0_/G_1_ phase of cell cycle or by induction of apoptotic cell death. Additionally, 1,25(OH)_2_D_3_ also has an impact on angiogenesis, thereby limiting the invasiveness of cancer cells [6]. As the key component of the vitamin D metabolism process, VDR similarly participates in the regulation of cancer development. SNPs of *VDR* gene have been shown correlated with cancers of the breast, prostate, colon [10], ovarian [14, 15], melanoma [16] and other malignancies [10, 17, 18]. In more than 200 specific *VDR*–SNPs, the most frequently associated with carcinogenesis are FokI (rs2228570), BsmI (rs1544410), TaqI (rs731236), ApaI (rs7975232) and Cdx2 (rs11568820) [10, 19, 20], yet the results are inconsistent. In contrast to the findings that *VDR* gene polymorphisms seem not related to the esophageal adenocarcinoma (EAC) risk development [21], we previously showed a significantly increased risk of esophageal squamous cell carcinoma associated with *VDR* rs2107301 T>C polymorphism among patients who were drinking [13]. Therefore, in this study, we sought to verify our hypothesis that SNPs in *VDR* gene is related to GCA since it occurs in the anatomical vicinity of esophagus. Similarly, none of the four polymorphic sites was associated with the change of susceptibility to GCA, but a remarkable increased risk of GCA was found among alcohol drinking but younger patients (<60 years of age) who carried *VDR* rs1989969 C>T genotype.

Previous studies have shown the correlation of several SNPs with GCA (summarized in [22]). PLCE1 (rs2274223) A>G SNP causes a missense variation in the protein phospholipase-Cε-1, which generates two critical messengers [inositol 1,4,5-triphosphate (IP3) and diacylglycerol (DAG)], thereby affecting cell growth, differentiation and gene expression [23]. Interestingly, vitamin D [(1,25(OH)_2_D_3_] – VDR signaling pathway also stimulates the IP3 and DAG generation via phospholipase C [24], which may probably underlie its correlation with GCA. Other genetic variants significantly associated with the risk of GCA included PRKAA1 (rs13361707), IL1B (rs16944), TNF (rs1800629) and MDM2 (rs2279744) [22], involving the signal transduction, inflammation, apoptosis aspects. However, the connections between these SNPs with our findings remain obscure.

As compared with the homozygote for the common allele, men who were homozygote for the rare allele for *VDR* rs2107301 have higher risk of prostate cancer [25]. In contrast, *VDR* rs2107301 was not associated with GCA in either single locus analyses or the stratified analyses in the current study. Instead, we demonstrated that *VDR* rs1989969 C>T polymorphism increased the risk of GCA among younger patients or alcohol drinkers, exemplifying the significance of the environment and genetic risk factors interact and both contribute to the carcinogenesis.

Our study showed the *VDR* T_rs2107301_T_rs2228570_C_rs1989969_G_rs11568820_ haplotype was associated with a significantly reduced risk of GCA, which indicated that polymorphism in single locus might not significantly modify the risk of cancer. The chain effect lying in different loci leads to a more profound impact which could regulate the risk of cancer.

Between ethnic groups, the frequencies of genetic polymorphisms do vary. In our study, the allele frequency of *VDR* rs1989969 was 0.323 in 608 control subjects, which is consistent with that in the Chinese Han (0.330) in the SNP Database, but lower than that of African (0.510) and Caucasian (0.410) population (http://www.ncbi.nlm.nih.gov/SNP).

Considering *VDR* rs1989969 C>T mutant alleles in the control group, ORs, GCA samples and control samples, the power of our analysis (α= 0.05) was 0.999 in 330 GCA cases and 608 controls with an OR of 2.05 in age<60 subgroup, and 0.983 with an OR of 1.78 in the drinking subgroup (PS, version 3.0, 2009, available at http://biostat.mc.vanderbilt.edu/twiki/bin/view/Main/PowerSampleSize).

We acknowledge that there are several limitations in the current study: First, restrained by the moderate sample size and lack of a validation cohort, the statistical power of our study was limited. Larger studies in multiple ethnical populations and various geographic locations are demanded to confirm the associations reported in our study. Second, the genetic effects of *VDR* polymorphisms on GCA susceptibility are probably caused by linkage disequilibrium (LD) with several functional variations within the *VDR* gene or with other closely linked genes. The SNPs we chose to study may not serve as a comprehensive representative of all the genetic variability of *VDR*, which entails further studies clarifying the genetic mechanism of GCA carcinogenesis by fine-mapping the susceptible region of the variants. Third, the study subjects recruited were from hospitals in the east part of China with same ethnicity, which may compromise its representativeness of the general population for potential inherited bias. Last but no least, the biological effects of VDR rs1989969 C>T polymorphism on VDR function and the downstream signaling cascade remain unclear. Located on the second intron of VDR, rs1989969 may probably cause an alternative RNA splicing on VDR mRNA, thereby regulating the VDR protein function. Yet this speculation demands further investigations.

In conclusion, the GCA is associated with a variety of factors including gene, environment and life-style. Our findings that the increased risk of GCA was found among alcohol drinking and younger patients (<60 years of age) who carried *VDR* rs1989969 C>T genotype and the reduced risk of GCA for man with *VDR* T_rs2107301_T_rs2228570_C_rs1989969_G_rs11568820_ haplotype, should be interpreted with much caution. Further larger studies in multiple ethnical populations and various geographic locations are needed to verify our preliminary results.

## MATERIALS AND METHODS

### Ethical approval of the study protocol

This hospital-based case-control study was approved by the Review Board of Jiangsu University (Zhenjiang, China). We have complied with the World Medical Association Declaration of Helsinki regarding ethical conduct of research involving human subjects and/or animals. All subjects provided written informed consent to be included. Each participant agreed to donate 2ml of peripheral venous blood for the research project, which was performed by skilled nurses under strict aseptic condition to minimize potential risks on subjects’ well being.

### Study subjects

The study included a total of 938 subjects. 330 patients with GCA were consecutively recruited from the Affiliated People's Hospital of Jiangsu University and Affiliated Hospital of Jiangsu University (Zhenjiang, China) between October 2010 and December 2012. The exclusion criteria were patients who previously had cancer, any metastasized cancer, radiotherapy or chemotherapy. The 608 controls were patients without cancer frequency-matched to the cases with regard to age (±5 years) and sex recruited from the two hospitals mentioned above during the same time period. Most of the controls were admitted to the hospitals for the treatment of trauma. Gender and age distribution had no significant difference between the case group and the control group, respectively.

Experienced and well-trained personnel interviewed each study subject with a pretested questionnaire. Demographic data and related risk factors were collected. 2mL samples of venous blood were collected from each subject with consent. Individuals who smoked one cigarette per day for ≥1 year were defined as “smokers”. Subjects who consumed ≥3 alcoholic drinks a week for >6 months were considered to be “alcohol drinkers”.

### Isolation of DNA and genotyping by ligation detection reaction

Blood samples from patients and controls were collected using vacuum blood tube with Ethylene Diamine Tetraacetic Acid (EDTA). Genomic DNA was isolated from whole blood by using QIAamp DNA Blood Mini Kit (Qiagen, Berlin, Germany). Gene polymorphisms were analyzed by the ligation detection reaction (LDR) method with technical support from the Biowing Applied Biotechnology (Shanghai, China). 10% of the total samples were randomly selected to repeated analyses in order to maximize the probably error of the genotyping results and improve quality control.

### Statistical analyses

Statistical analyses were performed using SPSS17.0 Statistical Package (2007, SPSS Inc., Chicago, IL). Hardy-Weinberg equilibrium for genotypes was tested by goodness-of-fit χ^2^ in control group. The distribution of *VDR* rs2107301 T>C, rs2228570 C>T, rs1989969 C>T and rs11568820 G>A genotypes was performed using the *chi*-square (χ^2^) test to examine statistical differences between patients and controls. The associations between these four SNPs and risk of GCA were estimated by computing the Odds ratios and confidence intervals (95%) using logistic regression analyses. Crude ORs and adjusted ORs when adjusting for age, sex, smoking and drinking status were also computed by using logistic regression analyses. Bilateral probability tests were taken, *p* value <0.05 on behalf of the difference was statistically significant.

## Abbreviations

VDR: vitamin D receptor
GCA: gastric cardiac adenocarcinoma
LD: linkage disequilibrium
OR: odds ratio
CI: confidential interval
SNPs: single-nucleotide polymorphisms
